# Modeling of hydrogen/deuterium dynamics and heat generation on palladium nanoparticles for hydrogen storage and solid-state nuclear fusion

**DOI:** 10.1016/j.heliyon.2015.e00057

**Published:** 2016-01-07

**Authors:** Katsuaki Tanabe

**Affiliations:** Department of Chemical Engineering, Kyoto University, Kyoto University-Katsura, Nishikyo-ku, Kyoto 615-8510, Japan

**Keywords:** Chemical engineering, Energy engineering, Materials science, Nanotechnology

## Abstract

We modeled the dynamics of hydrogen and deuterium adsorbed on palladium nanoparticles including the heat generation induced by the chemical adsorption and desorption, as well as palladium-catalyzed reactions. Our calculations based on the proposed model reproduce the experimental time-evolution of pressure and temperature with a single set of fitting parameters for hydrogen and deuterium injection. The model we generated with a highly generalized set of formulations can be applied for any combination of a gas species and a catalytic adsorbent/absorbent. Our model can be used as a basis for future research into hydrogen storage and solid-state nuclear fusion technologies.

## 1 Introduction

Micro- and nanoscale palladium (Pd) composites are known for their promise in hydrogen storage applications [1, 2, 3, 4, 5, 6, 7, 8, 9]. The possibility of realizing compact nuclear-fusion reactors with deuterium (D_2_) utilizing such palladium-based materials has been also investigated [10, 11, 12,13, 14, 15]. In order to make a detailed analysis and provide explanations for the experimental data for such molecular adsorption process that are accompanied by chemical reactions, the establishment of physical-chemical models is necessary. For the elementary nuclear reactions, a variety of reaction models have been proposed [16, 17, 18]. However, to the best of our knowledge, there are no reports on an exclusive process model that includes both the physical molecular transportation processes and the subsequent chemical reactions. These are necessary to reproduce the macroscopically observable experimental parameters, such as the pressure and temperature inside the reactors. In this study, we propose a model to describe the dynamics and heat generation in the H_2_/D_2_–Pd systems. This model provides a basis in these research areas for the evaluation and optimization of the operational performance with respect to the system structures and reaction conditions. The simple theoretical model we have developed reproduces the experimental time-evolution of the pressure and temperature observed in H_2_–Pd and D_2_–Pd systems with respect to the chemical adsorption and desorption of hydrogen and deuterium on Pd nanoparticles.

## 2 Theory and calculations

The model of the time-evolution of the Pd surface and inner coverage by H and D atoms is formulated by simplifying the phenomenon of adsorption and desorption of the gas-phase species on and in the Pd nanoparticles. In this study, we model the experimental results of H_2_ and D_2_ injection into a composite material containing Pd nanoparticles [2] reported in Refs. [13, 14]. For modeling, we assume a Langmuir-type adsorption–desorption mechanism. H_2_ molecules are known to first weakly physisorb onto Pd surface by van der Waals force and then strongly chemisorb there through dissociation into H atoms [19]. A first-order, all-inclusive rate equation on the H_2_/D_2_ gas pressure for adsorption and absorption is however assumed for simplicity, while the dissociative adsorption rate on Pd experimentally follows the square-root law at very high temperatures and low pressures [20]. The overall absorption rate can generally be a linear combination of a higher-order surface adsorption/desorption term on the density of H/D-vacancy sites and a first-order Pd-internal diffusive H/D atomic transport term [19]. We however adopt a first-order adsorption-absorption-inclusive rate equation for simplicity to introduce a prototype model in this work, while the H/D adsorption kinetics is actually not quite trivial as potentially associated with three or even more vacancy sites [21, 22] and thus would be hardly formulated in a clear form. We define the storage filling factor, $\xi_{a}$, as the ratio of the amount of adsorbed or absorbed atoms to their saturation storage level, i.e., 0 (vacancy, no storage) < $\xi_{a}$ < 1 (full storage). $\xi_{a}$ corresponds to the surface coverage ratio for the surface-adsorption-only cases represented by the Langmuir adsorption model. We also define a dimensionless parameter, $\xi_{gas}$, representing the amount of gas-phase hydrogen or deuterium in the reactor chamber normalized by the saturation amount of hydrogen or deuterium adsorbed or absorbed on (or in) the Pd nanoparticles. We then write the adsorption rate, including absorption into the nanoparticles for the context in this paper, of the gas species onto and into the Pd nanoparticles, *R_a_*, as

(1) $$R_{a}=k_{a}\left(1-\xi_{a}\right)\xi_{gas}$$

where *k_a_* is the adsorption-and-absorption rate constant. Similarly the desorption rate *R_d_* of hydrogen or deuterium on or in the Pd nanoparticles out into the atmosphere of the reactor chamber can be written as

(2) $$R_{d}=k_{d}\xi_{a}$$

where *k_d_* is the desorption rate constant. Note that we neglect the dependence of the adsorption and desorption rate constants on temperature because of the relatively small temperature range (25–70 °C) measured in the experiments. Also, we assume a sufficiently dilute gas atmosphere regime inside the reactor chamber for both H_2_ and D_2_ so that the desorption rate of the H_2_ and D_2_ species from Pd can be regarded as independent of their gas-phase concentrations or pressures (i.e., the dependence of the desorption rate on the gas concentration or pressure is negligible). The total rate of the H and D storage evolution on or in Pd is therefore:

(3) $$\frac{d\xi_{a}\left(t\right)}{dt}=R_{a}\left(t\right)-R_{d}\left(t\right)=k_{a}\left\{1-\xi_{a}\left(t\right)\right\}\xi_{gas}\left(t\right)-k_{d}\xi_{a}\left(t\right)$$

where *t* is the time elapsed. For molecular mass balance:

(4) $$\xi_{gas}\left(t\right)=J_{in}t-\xi_{a}\left(t\right)$$

where *J_in_* is the H_2_ or D_2_ gas injection flow rate into the reactor chamber, assuming a constant injection rate equivalent between H_2_ and D_2_ in the experiments. Eqs. (3) and (4) give:

(5) $$\frac{d\xi_{a}\left(t\right)}{dt}=k_{a}\left\{1-\xi_{a}\left(t\right)\right\}\left\{J_{in}t-\xi_{a}\left(t\right)\right\}-k_{d}\xi_{a}\left(t\right)$$

We solved this differential equation Eq. (5) numerically by the forward Euler method with the initial condition:

(6) $$\xi_{a}\left(t=0\right)=0$$

to calculate $\xi_{gas}$, which is then converted into the pressure in the reactor chamber, *P(t)*, via:

(7) $$P\left(t\right)=P_{0}\xi_{gas}\left(t\right)$$

where *P_0_* is a constant corresponding to the pressure when the number of the gas-phase atoms (twice the number of the molecules) equals the saturation number of atoms on or in the Pd nanoparticles (n.b., this situation has no particular physical significance) and a free parameter, just at the point of this work, in the following fitting to the experimental data.

To model the time-evolution of the temperature of the Pd-based composite material, we have formulated Eq. (8). This equation represents the heat balance to determine the temperature evolution caused by heat generation and dissipation.

(8) $$C\frac{dT}{dt}=Q_{a-d}+Q_{reac}-Q_{diss}$$

In Eq. (8), *T*, *C*, *Q_a-d_*, *Q_reac_* and *Q_diss_* are the temperature of the Pd-based specimen, the heat capacity of the Pd-based composite material, the heat generation induced by the adsorption and desorption heat of hydrogen and deuterium with Pd, the heat generation induced by the nuclear reaction of deuterium on (or in) Pd, and the heat dissipation out from the Pd composite, respectively. For each heat generation or dissipation term, we assume that:

(9) $$Q_{a-d}\propto \frac{d\xi_{a}}{dt}$$

(10) $$Q_{reac}\propto {\xi_{a}}^{2}$$

and

(11) $$Q_{diss}\propto T-T_{0}$$

where *T_0_* is the ambient temperature of the location in which the reactor chamber is located, and is assumed to be 25 °C. Eq. (9) simply accounts for the adsorption or desorption enthalpy. For Eq. (10), we assume the most commonly accepted two-body D–D nuclear fusion reaction [13, 16, 17, 18]. We assume heat dissipation in the form of Eq. (11) in accordance to Fourier's law for heat transportation, where the contribution of radiation is negligible. We then formulate:

(12) $$\frac{dT\left(t\right)}{dt}=h_{a-d}\frac{d\xi_{a}\left(t\right)}{dt}+h_{reac}\left(T\right){\left\{\xi_{a}\left(t\right)\right\}}^{2}-h_{diss}\left\{T\left(t\right)-T_{0}\right\}$$

from Eqs. (8), (9), (10), (11), where *h_a-d_*, *h_reac_*, and *h_diss_* are coefficients for the heat generation or dissipation by the chemical adsorption and desorption, deuterium nuclear reaction and conductive heat dissipation, respectively. In addition, we include the temperature-dependence of the reaction coefficient:

(13) $$h_{reac}\left(T\right)=h_{r0}\exp\left(-\frac{E_{a}}{k_{B}T\left(t\right)}\right)$$

in an Arrhenius-type form, where *h_r0_* is a reaction-rate constant, *E_a_* is the activation chemical potential for the reaction, and *k_B_* is the Boltzmann constant. We have numerically solved Eq. (12) with $\xi_{a}\left(t\right)$ calculated in the previous section and the initial condition:

(14) $$T\left(t=0\right)=T_{0}$$

to determine *T(t)*. The reaction-induced heat term, the second term on the right hand in Eqs. (8) and (12), is omitted for the calculation in the case of H_2_. The consumption of deuterium by the nuclear reaction is assumed to be negligible relative to the number of deuterium adsorbed on Pd because of the low probability of the nuclear reaction and the rapid compensation of newly generated D-vacancy sites on Pd by deuterium adsorption from the gas phase in the chamber.

## 3 Results and discussion

**Fig. 1:**
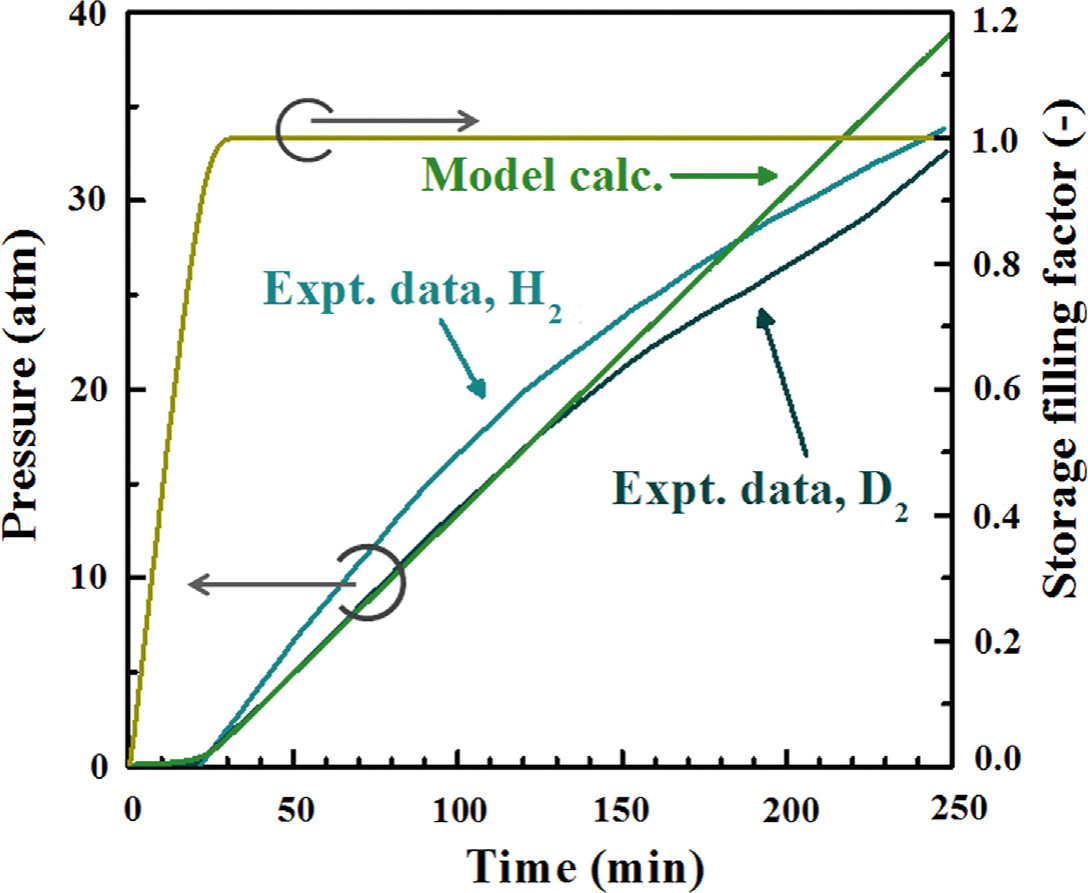
Experimental and calculated time-evolution of the pressure in the reactor chamber for the H_2_–Pd and D_2_–Pd systems. The experimental data is reproduced from Ref. [14] (public domain). Also plotted is the calculated time-evolution of the storage filling factor.

**Fig. 2:**
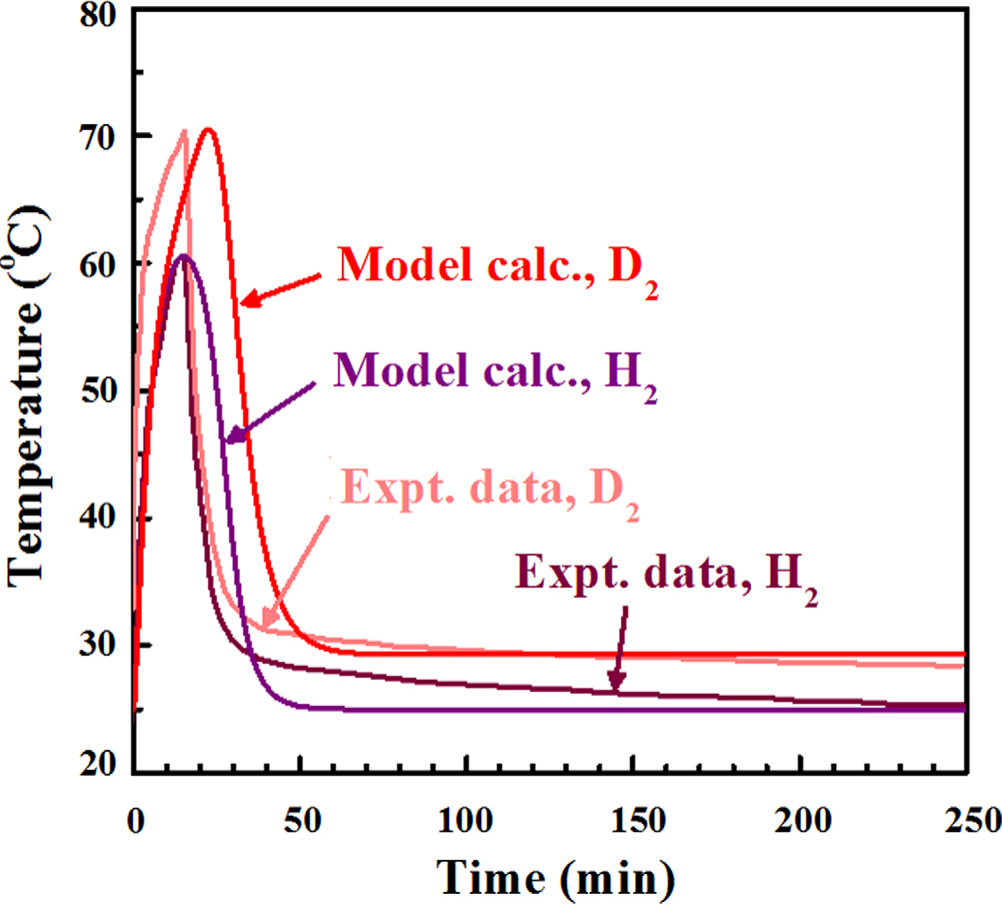
Experimental and calculated time-evolution of the temperature of the Pd-based composite specimen for the H_2_–Pd and D_2_–Pd systems. The experimental data is reproduced from Ref. [14] (public domain).

We have numerically solved the differential equations Eqs. (5) and (12) to calculate the time-evolution of the pressure *P(t)* and temperature *T(t)*. We then fitted these to an existing set of experimental data for the corresponding reaction system reproduced from Refs. [13, 14] (the subsequent, polished version of the team's earlier work in Ref. [10]). Fig. 1, Fig. 2 show our model calculation results for *P(t)* and *T(t)* fitted to the experimental data. The fitting parameters we used for the calculations are *k_a_* = 3.0 × 10^−2^ s^−1^, *J_in_* = 7.9 × 10^−4^ s^−1^, *k_d_* = 8.3 × 10^−6^ s^−1^, *P_0_* = 3.6 atm, *h_a−d_* = 1.7 × 10^2^ K, *h_diss_* = 3.3 × 10^−3^ s^−1^, *h_r0_* = 1.1 × 10^5^ K/s and *E_a_*/*k_B_* = 4.8 × 10^3^ K. As seen in Fig. 1, the time-evolution of the pressure in the reactor chamber is well reproduced by our simple model for both the case of H_2_ and of D_2_ injection. It should be noted that the calculated *P(t)* curve is identical for the H_2_ and D_2_ cases because we assume in this work that the physical constants *k_a_*, *k_d_*, *J_in_*, and *P_0_* are equivalent between hydrogen and deuterium.

The time-evolution behavior of the pressure seen for the experimental and theoretical curves in Fig. 1 is interpreted as follows. The gas pressure in the reactor chamber stays almost zero until the Pd material is nearly filled up by adsorption of hydrogen or deuterium. This occurs because the adsorption/absorption rate constant, *k_a_*, is significantly larger than the desorption rate constant, *k_d_*. The pressure then starts to rise once the adsorption/absorption of hydrogen or deuterium on and in the Pd nanoparticles reaches saturation. This phenomenal observation is also supported by the calculated *ξ_a_* temporal behavior, also plotted in Fig. 1. Similar behavior for the pressure evolution in H_2_–Pd and D_2_–Pd systems under vacuum has been experimentally observed [15].

Since we do not know the values of experimental parameters such as the reactor chamber volume and the gas injection flow rate, we used the procedure outlined above to handle the normalized dimensionless parameters for our calculations in this work. However, it would be more practical to conduct the calculations with absolute-valued parameters utilizing known experimental parameters. For example, an alternative formulation is:

(15) $$\frac{dn_{a}\left(t\right)}{dt}=k_{a}^{\prime }\left\{N_{site}-n_{a}\left(t\right)\right\}\left\{J_{in}^{\prime }\;t-n_{a}\left(t\right)\right\}-k_{d}n_{a}\left(t\right)$$

where *n_a_(t)* is the number of H or D atoms adsorbed or absorbed on the Pd-based composite, $k_{a}^{\prime }$ is an adsorption-desorption rate constant and

(16) $$k_{a}^{\prime }=\frac{k_{a}}{N_{site}}$$

*N_site_* is the number of sites on and in the Pd-based composite material that can store hydrogen and deuterium (i.e., the saturation number of H and D atoms to be adsorbed or absorbed on Pd), and $J_{in}^{\prime }$ is given by:

(17) $$J_{in}^{\prime }=N_{site}J_{in}$$

and is the absolute injection flow rate of the H_2_ or D_2_ gas into the reactor chamber, which is thus well known and controllable in experiments. Also, *N_site_* can be relatively straightforwardly determined for experimental Pd materials, for example, by Brunauer-Emmett-Teller adsorption measurements [23].

The temperature evolution for both the cases of hydrogen and deuterium are reproduced well by our model calculation as seen in Fig. 2. Significantly, we used an identical set of the fitting parameters between the hydrogen and deuterium cases, except for *h_r0_*, which was set to be zero for the H_2_–Pd system because we assume that no nuclear reactions occur for hydrogen, leading to the well-fitted results displayed in Fig. 2. The time-evolution behavior of the temperature, seen in Fig. 2, can be interpreted generally as follows: Firstly, the temperature rapidly rises due to the heat generation induced by the chemisorption of H and D onto the Pd material, as well as the nuclear reaction particularly for the case of D_2_ injection. Then the temperature plateaus and starts to slowly decrease once the adsorption sites on the Pd nanoparticles are totally filled up by H or D. This occurs because there is no more heating caused by chemisorption and heat is lost by dissipation caused by the temperature difference between the Pd material and the surrounding environment. In the case of D_2_, we see a discrepancy in time between the experimental data and the calculated data for the early-stage temperature peaks. This discrepancy can be recognized as the technical limit of our present simplistic model. Improving the agreement between experimental and calculated data is a project for the continued development of a more detailed model. The calculations based on our simple model reproduced, to a remarkable degree, the temperature difference between the hydrogen and deuterium cases at the peaks (∼ 10 °C), apparently due to the influence of the reaction term in Eq. (12), as well as at the tails in the long-time region (∼ 5 °C), as seen in Fig. 2. In the case of deuterium, the temperature is maintained around 30 °C, above the ambient temperature, because of the balance between Fourier's heat dissipation out from the Pd sample and heat generation presumably caused by the deuterium fusion reaction. This temperature behavior is also well reproduced by our model. Incidentally, although we adopted the most common quadratic formulation for the nuclear fusion reaction in this study, it should be noted that there have been proposals for cubic and quartic reaction models [11, 18].

Thus, the model we propose reproduces well both the pressure and temperature evolution of the experimental results for the H_2_/D_2_–Pd gas-solid-phase chemical reaction system for both hydrogen and deuterium by using an identical set of parameters: validating our model. The physical constant parameters, such as those in Eqs. (5) and (12), can be determined by by collecting experimental data under varied conditions. Using these new parameters, we can then construct more realistic models and hence direct the practical uses for the devices and methods to improve their performance, based on our model. It should be noted that since our formulation presented in this paper is highly generalized and material-independent, our model can potentially be applied for any kind of reaction system with a gas species and a micro/nanostructured absorbent or adsorbent. The model presented in this paper can act as a basis of further research into the realization and optimization of high-performance hydrogen storage and solid-state nuclear fusion technologies.

## 4 Conclusions

We have proposed a simple model for the emerging H_2_–Pd and D_2_–Pd nanostructured chemical systems that accounts for the adsorption and desorption processes as well as the Pd-catalyzed reactions. Our calculations based on the model and using the same set of fitting parameters have well reproduced the experimental evolution of pressure and temperature in the cases of both hydrogen and deuterium in corresponding systems. Thus, the validity of our model is demonstrated. The model presented in this paper can act as the basis for further research, and can be used as a powerful tool to analyze the chemical phenomena occurring inside the H_2_/D_2_–Pd reaction systems. This will support the future development and improvement of such practical energy-storing and energy-generating devices. Furthermore, our highly generalized numerical model can potentially be applied for any sort of adatoms and adsorbents, not only for the specific reaction system adopted as a case study in this work.

## Declarations

### Author contribution statement

Katsuaki Tanabe: Conceived and designed the study; Analyzed and interpreted the data; Wrote the paper.

### Funding statement

This work was partially supported by the JFE Steel Corporation 21st Century Foundation, the Japan Society for the Promotion of Science (JSPS), and the Ministry of Education, Culture, Sports, Science and Technology-Japan (MEXT).

### Competing interest statement

The authors declare no conflict of interest.

### Additional information

No additional information is available for this paper.
